# MRI-Based Quantification of Magnetic Susceptibility in Gel Phantoms: Assessment of Measurement and Calculation Accuracy

**DOI:** 10.1155/2018/6709525

**Published:** 2018-07-30

**Authors:** Emma Olsson, Ronnie Wirestam, Emelie Lind

**Affiliations:** Department of Medical Radiation Physics, Lund University, Skåne University Hospital Lund, 22185 Lund, Sweden

## Abstract

The local magnetic field inside and around an object in a magnetic resonance imaging unit depends on the magnetic susceptibility of the object being magnetized, in combination with its geometry/orientation. Magnetic susceptibility can thus be exploited as a source of tissue contrast, and susceptibility imaging may also become a useful tool in contrast agent quantification and for assessment of venous oxygen saturation levels. In this study, the accuracy of an established procedure for quantitative susceptibility mapping (QSM) was investigated. Three gel phantoms were constructed with cylinders of varying susceptibility and geometry. Experimental results were compared with simulated and analytically calculated data. An expected linear relationship between estimated susceptibility and concentration of contrast agent was observed. Less accurate QSM-based susceptibility values were observed for cylindrical objects at angles, relative to the main magnetic field, that were close to or larger than the magic angle. Results generally improved for large objects/high spatial resolution and large volume coverage. For simulated phase maps, accurate susceptibility quantification by QSM was achieved also for more challenging geometries. The investigated QSM algorithm was generally robust to changes in measurement and calculation parameters, but experimental phase data of sufficient quality may be difficult to obtain in certain geometries.

## 1. Introduction

An object in an external magnetic field will become magnetized to a degree that is determined by the magnetic susceptibility of the object. The local magnetic field inside and in the surroundings of the object will thus depend on the magnetic susceptibility in combination with the geometry/orientation of the object being magnetized. In susceptibility-weighted magnetic resonance imaging (SWI), the local magnetic field distribution is explored to enhance image contrast and to improve the visibility of various structures on the basis of their magnetic susceptibility [1]. Additionally, quantitative susceptibility mapping (QSM) has developed into a promising method for calculating arbitrary magnetic susceptibility distributions from measured magnetic resonance imaging (MRI) phase data [2–4]. A more complete understanding of the phase behaviour in vivo may also require biophysical considerations of microstructural tissue anisotropy and magnetic susceptibility anisotropy [5].

In vivo, the magnetic susceptibility differs among tissue types and tissue regions, and it can thus be exploited as a source of contrast in MRI. For example, in reference to the cerebrospinal fluid (CSF), i.e., when the CSF magnetic susceptibility is set to 0 ppm, the globus pallidus, which is part of the basal ganglia, has a magnetic susceptibility of 0.105 ppm while white matter has a lower susceptibility of -0.030 ppm [6]. In addition to the effects of normal ageing [7], a number of conditions and processes can alter the magnetic susceptibility of tissue. For example, iron accumulation in inflamed myelin cells, as in a multiple sclerosis (MS) plaque, increases the susceptibility of the myelin [8]. Iron accumulation is also seen in other neurodegenerative diseases, for example, Alzheimer's and Parkinson's diseases [9]. The magnetic susceptibility is also dependent on the oxygen saturation level of blood, and the susceptibility increases with increasing levels of deoxyhemoglobin. Hence, venous blood will show a higher susceptibility than arterial blood, and quantification of magnetic susceptibility can thus be useful in estimations of oxygen extraction fraction (OEF) [10] and cerebral metabolic rate of oxygen (CMRO_2_) [11]. The change in susceptibility with deoxygenation can also be manifested, for example, in extravasated blood from an intracranial haemorrhage [12]. Quantitative measurements of the susceptibility could also be potentially useful to determine the concentration of an external MRI contrast agent (CA). Relaxivity-based CA quantification, which is the currently most common approach, is associated with several methodological complications in, for example, perfusion and permeability measurements using dynamic contrast-enhanced MRI (DCE-MRI) and dynamic susceptibility contrast MRI (DSC-MRI) [13]. Hence, more accurate CA concentration quantification in vivo would indeed be beneficial, and a few examples of dynamic contrast-enhanced QSM studies have been presented [14–16].

In QSM applications, quantification of magnetic susceptibility in absolute terms is becoming increasingly important, and extensive validation is thus warranted. A number of QSM reconstruction tools exist, and the process of systematically characterizing differences in accuracy between algorithms has recently been initiated by other groups [17–19]. In experimental evaluations, phantoms have the advantage of offering well-defined contents and geometries and constitute an important, though not complete, part of the validation process, and the present investigation serves as a supplement to previous investigations related to the accuracy of phase and susceptibility quantification [e.g., [20–25]]. In the present study, previously described in preliminary terms by Olsson (unpublished report) [26], the QSM approach was evaluated in gel phantoms with inserted cylinders containing known concentrations of gadolinium CA, to establish whether the QSM method can deliver accurate results with respect to quantitative magnetic susceptibility values in absolute terms. Measured phase values and the corresponding magnetic susceptibility estimates, calculated by an established QSM algorithm, were compared to values based on theoretical relationships. Various phantom designs and simulated susceptibility distributions, as well as different parameters and settings in the measurements and in the susceptibility calculation, were investigated in order to establish optimal settings and important sources of error in the attempts to produce accurate magnetic susceptibility maps.

## 2. Theory

### 2.1. The Dipole Field and the Magic Angle

A magnetic moment with magnitude *m*, pointing in the *z* direction, produces a magnetic flux density component *B*_*z*_:

(1) $$\begin{array}{c} B_{z}\propto \frac{m}{d^{3}}\left(\;3\;\cos^{2}\Theta -1\;\right), \end{array}$$

where* d* is the distance from* m* and Θ is the angle relative to the z-axis. The angle at which the factor (3 cos^2^⁡Θ − 1) equals zero is called the magic angle (i.e., approximately ±54.7° or 180°±54.7°). At the magic angle positions, the magnetic flux density component *B*_*z*_ will be zero independently of the magnitude of the magnetic moment.

### 2.2. Phase Shift and Magnetic Field

Variations in the local magnetic field with position* r* lead to differences in MRI resonance frequency and to subsequent phase-shift variations, and the MRI phase evolution* ϕ(r)* is given by

(2) $$\begin{array}{c} \phi \left(\;r\;\right)=\omega \left(\;r\;\right)\cdot TE=\gamma \cdot B\left(\;r\;\right)\cdot TE, \end{array}$$

where TE is the echo time. In order for the measured phase images to be useful, unwrapping and filtering of background field variations are required. The unwrapping can be accomplished by a region growing algorithm which identifies phase gradients that correspond to a difference by a multiple of 2*π* and subsequent addition or subtraction of 2*π* [27]. Filtering is needed because the unwrapped image usually contains a remaining background phase gradient over the entire image. This phase does not arise from the susceptibility distribution inside the object but from, for example, imperfect shimming or susceptibility sources outside the imaging volume.* Projection onto Dipole Fields *(PDF) [28, 29] is one method for background field removal that compares magnetic fields generated from magnetic dipoles inside and outside a region of interest. Other examples of filtering methods are* Laplacian Boundary Value *(*LBV*) [30] and* Regularization Enabled Sophisticated Harmonic Artefact Reduction for Phase data *(*RESHARP*) [31].

### 2.3. Cylindrical Objects

The internal (in) and external (ex) magnetic field alterations ΔB, caused by an infinitely long cylinder, are given by the following analytical expressions:

(3) ΔBin=Δχ63cos2⁡θ−1B0

(4) ΔBex=Δχ2a2ρ2sin2⁡θcos⁡2φB0

where Δ*χ* is the difference in susceptibility between the inside and the outside of the cylinder, *a* is the radius of the cylinder, *θ* is the angle between the direction of the B_0_ field and the cylinder axis, and *ρ* and *φ* are the cylindrical coordinates describing a point at distance *ρ* and at an angle *φ* relative to a point at the centre of the cylinder.

### 2.4. Magnetic Susceptibility and Magnetic Field

For more complicated geometries or shapes, the local field change caused by the introduction of an object in the external magnetic field can be described more generally [32, 33] and is often formulated as a convolution (denoted “⊗”) of the arbitrary susceptibility distribution with a dipole field kernel; i.e., the corresponding phase is given by

(5) $$\begin{array}{c} \Delta \phi \left(\;r\;\right)=\gamma \cdot TE\cdot \frac{3\cos^{2}\theta -1}{4\pi r^{3}}⊗\chi \left(\;r\;\right) \end{array}$$

where *r* and *θ* are spherical coordinates and *χ* denotes the magnetic susceptibility. The main idea of QSM is to extract the susceptibility distribution according to (5), using the information of the local magnetic field from the measured phase images. However, problems arise because the dipole kernel is zero at the magic angle. A convolution in real space represents a multiplication in k-space, and extracting the susceptibility distribution from (5) by deconvolution would therefore imply a division by zero at some coordinates in k-space which would, in principle, affect every point of the *χ(r)* solution in real space.

*Morphology Enabled Dipole Inversion *(MEDI) [29, 34–36] is a QSM reconstruction method, designed to solve the ill-posed inverse problem of resolving* χ(r)* according to (5). In the MEDI approach, the problem is formulated so that the difference between an estimated field map and the measured field map should be of the order of the noise level* ε*. This can be written as

(6) $$\begin{array}{c} {\left\|\;W\left(\;\delta -FT^{-1}\left(\;D\cdot FT\left(\;\chi \;\right)\;\right)\;\right)\;\right\|}_{2}\leq \varepsilon \end{array}$$

where *W* is a weighting matrix, *δ* is the measured field, and* D* is the representation of the dipole field in k-space. “*FT*” and “*FT*^−1^” denote the forward and inverse Fourier transform, respectively. Additionally, MEDI uses the fact that changes in susceptibility follow the morphological boundaries and that the susceptibility map therefore should have gradients in the same locations as the magnitude image [35].

In brief, the inverse problem is solved through an iterative process. An initial guess is made for the susceptibility distribution. Convolving this with the dipole kernel gives an estimated field map. The estimated field map is compared to the measured field map, i.e., the phase image, and the difference, the error, is used to update the initial guess. The updated susceptibility distribution is then used as input when this procedure is repeated. Iterations are made until the result fulfils the requirements. A regularization parameter *λ* determines how much magnitude versus phase image information is prioritized. The Lagrange multiplier method is used to reformulate the problem in (6) as a minimization of a cost function [35].

## 3. Materials and Methods

### 3.1. Phantom Design

In order to evaluate the QSM method with respect to phase measurement as well as mathematical reconstruction, three different phantoms were constructed. Thin-walled plastic cylinders were filled with a paramagnetic gadolinium (Gd) contrast agent solution (Dotarem, Guerbet, France). The employed plastic material and the low thickness of the cylinder walls (of the order of 100 *μ*m) imply that the susceptibility effects created by the cylinders should be negligible. The cylinders were sealed and glued onto the inside of a larger container. The container with cylinders was subsequently filled with agarose gel doped with a small amount of nickel in the form of nickel(II)nitrate hexahydrate, Ni(NO_3_)_2_·6H_2_O. The gel was designed according to a locally developed preparation routine using, in this study, 1% agarose and 0.24 mM Ni^2+^ [37]. The susceptibility of the gel was calculated using Wiedemann's additivity law for the susceptibility of mixtures, i.e., *χ* = *p*_1_*χ*_1_ + *p*_2_*χ*_2_ + ⋯*p*_*n*_*χ*_*n*_, where *p*_*n*_ is the concentration of substance *n* [38].

The purpose of the contrast agent was to obtain a controlled increase of the susceptibility inside the cylinders to achieve a difference in susceptibility between the cylinders and the background, resembling different compartments in the human body (including cases of injected external contrast agent, for example, for the purpose of perfusion imaging).  Table 1 shows the theoretical absolute values used for the susceptibility of water and nickel, as well as the most commonly reported value of the molar susceptibility for gadolinium (used as a reference value in this study [39]). The calculated susceptibility values for the gel and the 0.5 mM gadolinium solution are also included.

- In the first phantom design, cylinders with 5 mm diameter, filled with 0.5 mM Gd solution, were positioned at five different angles relative to the main magnetic field (approximately 0, 30, 55, 75, and 90°). The actual angles were measured in the resulting images.
- The second phantom design consisted of 5 mm diameter cylinders in parallel, with varying concentrations of Gd contrast agent, i.e., [0, 0.2, 0.4, 0.6, 0.8, 1, 2, 4, 6, 8, 10] mM.
- In the third phantom, cylinders in parallel, containing 0.5 mM Gd solution with diameters of [2, 2.6, 4.7, 5, 7.4, 9, 10.8] mm, were used.

### 3.2. Measurements

Measurements were carried out at room temperature on a 3T MRI unit (Magnetom Trio, Siemens Healthcare GmbH, Erlangen, Germany) using an imaging protocol described in Table 2. The parameters were selected according to the recommendations for QSM of human brain given by the* Cornell MRI Research Lab* [40]. A multi-TE gradient echo sequence was used, because a single TE acquisition is regarded not to be sufficient for deriving the magnetic field from the phase, due to an offset in the magnetic field depending on the conductivity of the material. The phase shifts reported below correspond to the difference in phase between two subsequent TEs, i.e., to a time period ΔTE (*cf.*Table 2), and are based on multiecho data [34, 39, 41]. In the evaluation process, this protocol was subsequently altered to accomplish measurements with isotropic voxels, different spatial resolutions, and shorter TE. The different parameter changes are described more in detail below.

*Spatial Resolution*. Measurements were performed with varying spatial resolution (altered matrix size and fixed volume of interest). A FOV of 205×205 mm^2^ and an excited slab of 32 mm were used. The matrix sizes used were 64×64, 128×128, 256×256, and 512×512, corresponding to isotropic voxels with sides 3.2, 1.6, 0.8, and 0.4 mm, respectively.

*Volume Coverage*. Measurements in which only the number of slices was varied were carried out, implying varied volume of interest. The voxel size was 0.8×0.8×0.8 mm^3^ and the number of slices was 40, 60, 104, and 144. The phantom with different diameters was used, placed with the cylinders perpendicular to the main magnetic field.

### 3.3. Image Processing and QSM Calculation

For postprocessing of the measured images, a MEDI MATLAB code package for QSM, from* Cornell MRI Research Lab* [40], was employed. Magnitude and phase images, one set for each TE, were obtained from the MRI experiments. The phase images were unwrapped [27] and subsequently filtered with the PDF approach [28, 29]. The phase images were also masked before they were supplied to the MEDI algorithm, using a threshold approach based on information from the magnitude image, to define the object region to be included in the susceptibility calculation.

### 3.4. Variations in Postprocessing and QSM Calculation Procedures

*Filtering Method*. Different methods for phase background removal were compared, i.e., “Projection onto Dipole Fields (PDF)” [28, 29], “Laplacian Boundary Value (LBV)” [30], and “Regularization Enabled Sophisticated Harmonic Artefact Reduction for Phase data, (RESHARP)” [31].

*Variation of λ*. The susceptibility images were calculated using *λ* settings 1, 10, 100, 1000, 10 000, and 50 000. 

*Zero Padding*. Zero padding, to potentially reduce artefacts, was performed in the spatial domain by padding the matrix symmetrically with 200 zeros in all three dimensions.

### 3.5. Simulation

**Figure 1:**
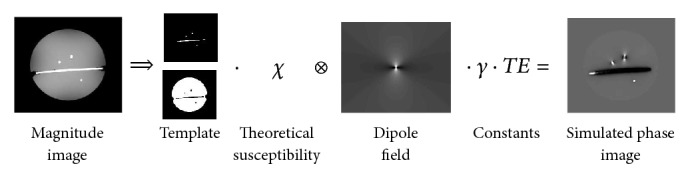
Illustration of the construction of a simulated phase image.

A simulated set of phase images was constructed by creating a template based on the magnitude images that distinguishes between cylinders and gel. Artificial susceptibility images were then constructed by assigning the theoretical values (*cf.*Table 1) of the susceptibility for agarose gel and gadolinium solution to the respective pixels. From the artificial susceptibility images, a set of simulated phase images was calculated using (5). This set of simulated phase images was then used as input to the MEDI algorithm and simulated QSM maps were obtained. The construction of the simulated phase image is illustrated in Figure 1.

### 3.6. Image Analysis

**Figure 2:**
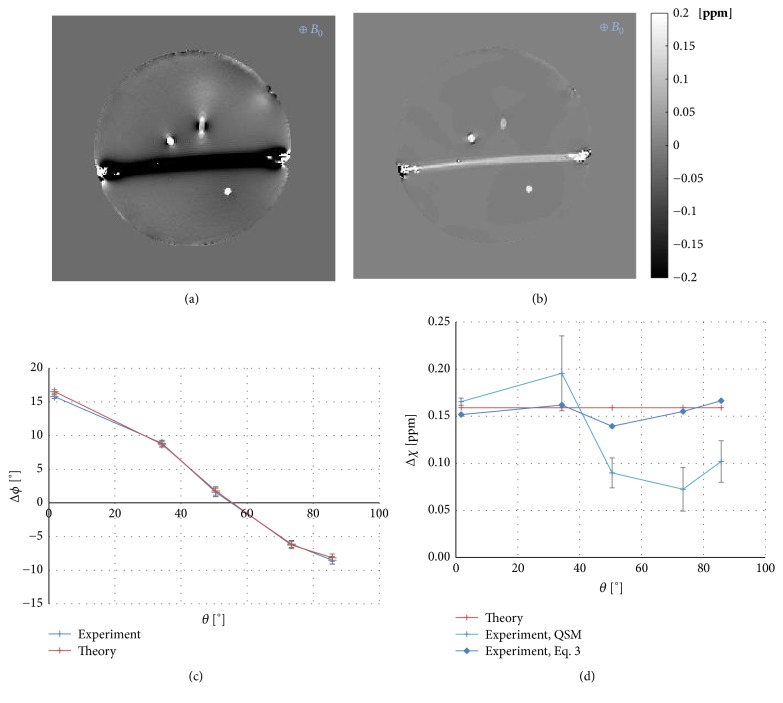
(a) Phase image and (b) the corresponding calculated susceptibility image of the phantom with varying angles of the cylinders (slice 17 of 48). The graphs in (c) and (d) show theoretical as well as experimental phase and susceptibility in the cylinders as a function of the angle relative to the main magnetic field. (c) Analytically calculated phase values (based on the infinite-cylinder approximation and the reference values in Table 1) and the corresponding measured phase values. (d) Expected theoretical magnetic susceptibility values (based on the reference values in Table 1) as well as the estimates based on experimental phase data, i.e., employing the infinite-cylinder approximation as well as the QSM algorithm. Error bars correspond to the standard deviation within the region of interest for the experimental QSM data.

Experimental as well as simulated phase and QSM images were evaluated by measuring the value of interest (mean and standard deviation) in ROIs placed in the cylinders. In the output data from the MEDI software, the background gel region was assigned values which were very close to zero (*cf.*Figure 2), and the background gel thus served as a zero reference (with known susceptibility according to Table 1). Experimental values were compared with the corresponding theoretical and/or simulated values. The calculations of theoretical phase, displayed for the phantom with 0.5 mM Gd cylinders of varying angles and for the phantom with varying Gd contrast agent concentrations, were based on (3) under the assumption that the respective magnetic susceptibility differences Δ*χ* were known, based on the reference values in Table 1. Conversely, (3) can be used to calculate an unknown magnetic susceptibility difference, based on measured phase, and such calculated Δ*χ* estimates, based on measured phase and the infinite-cylinder approximation of (3), are also, for completeness, included in the results.

## 4. Results

### 4.1. Cylinder Angle

Figure 2 shows (a) a phase image and (b) a corresponding susceptibility image of the phantom with cylinders of varying angles relative to the B_0_ field. So-called blooming effects in the phase image, related to the properties of a dipole field, are seen around cylinders not oriented parallel to the main magnetic field. Some unwanted residues of this blooming effect can be seen in the susceptibility image. In Figure 2(c), analytically calculated phase values, based on the infinite-cylinder approximation (see (3)) and the assumption of known reference values of magnetic susceptibility (Table 1), are compared with the measured phase data for cylinders at different angles.  Figure 2(d) shows the expected magnetic susceptibility values (based on the reference values in Table 1), as well as the corresponding results based on experimental phase data, i.e., employing the infinite-cylinder approximation (see (3)) as well as the QSM algorithm. The phase inside the cylinders corresponded well with the theoretical values, and, accordingly, the infinite-cylinder approximation yielded quite reasonable susceptibility estimates based on experimental phase data. However, the QSM calculation returned susceptibility values that deviated considerably from the expected values, for angles larger than the magic angle.

### 4.2. Concentration of Gadolinium Solution

**Figure 3:**
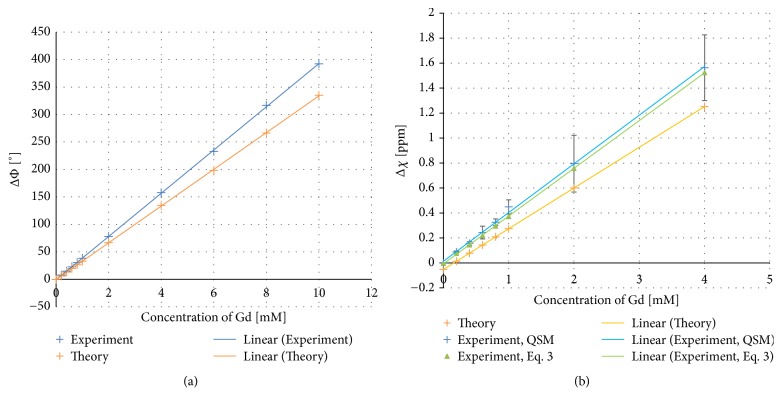
Phase and magnetic susceptibility as a function of the concentration of gadolinium contrast agent. (a) Analytically calculated phase (based on the infinite-cylinder approximation and the reference values in Table 1) and measured phase. (b) Expected theoretical magnetic susceptibility values (based on the reference values in Table 1) as well as the estimates based on experimental phase data, i.e., employing the infinite-cylinder approximation as well as the QSM algorithm. For the three highest concentrations in (a), the phase was manually unwrapped. Error bars correspond to the standard deviation within the region of interest for the experimental QSM data.

An expected linear dependence, for both phase and susceptibility, on the concentration of gadolinium was observed (Figure 3). The QSM algorithm yielded a measured slope of 390 ppm/M, which was slightly higher than the corresponding experimental slope based on the infinite-cylinder approximation (382 ppm/M) (Figure 3(b)). Insufficient phase unwrapping was observed at high concentrations, and, for the phase results, this was compensated for manually by simply adding 2*π* to the extracted numerical values. It was, however, not possible to evaluate the QSM output for concentrations above 4 mM.

### 4.3. Cylinder Diameter

**Figure 4:**
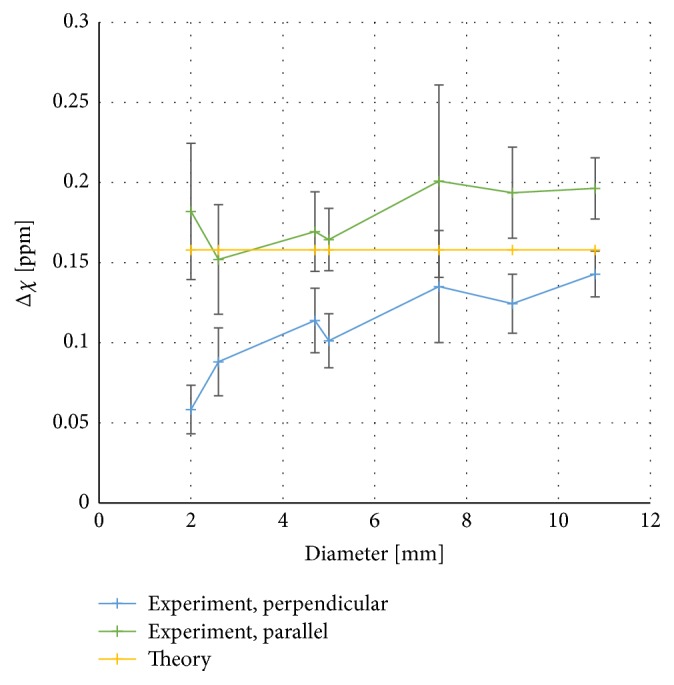
Measured susceptibility estimated from the QSM algorithm as a function of cylinder diameter. Results for cylinders parallel and perpendicular to the main magnetic field are compared with the theoretical value. Error bars correspond to the standard deviation within the region of interest for the experimental data.

QSM-based susceptibility estimates for the seven cylinders of various diameters, at parallel and perpendicular orientations, are presented in Figure 4.

### 4.4. Variation of Measurement Parameters

**Figure 5:**
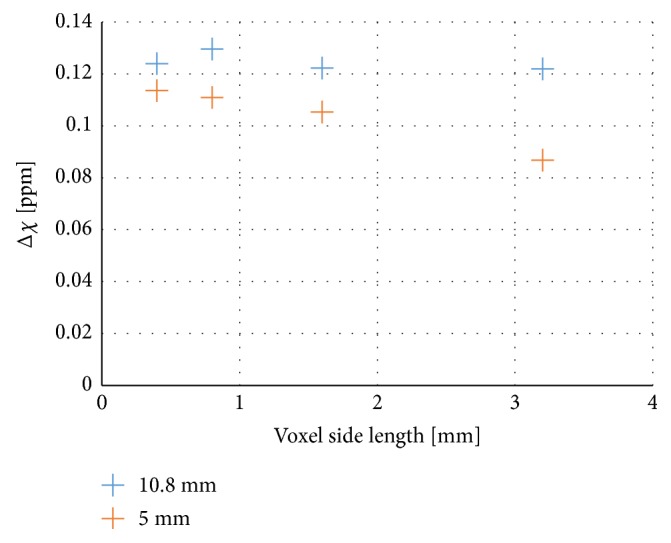
Measured susceptibility estimates from the QSM algorithm in the centres of the 10.8 mm and 5 mm diameter perpendicularly oriented cylinders, for different isotropic spatial resolutions.

*Spatial Resolution*. The phantom with different diameters was measured, with the cylinders oriented perpendicular to the main magnetic field, at different spatial resolutions. The susceptibilities in the 5 mm and 10.8 mm cylinders are presented as a function of pixel size in Figure 5.

**Figure 6:**
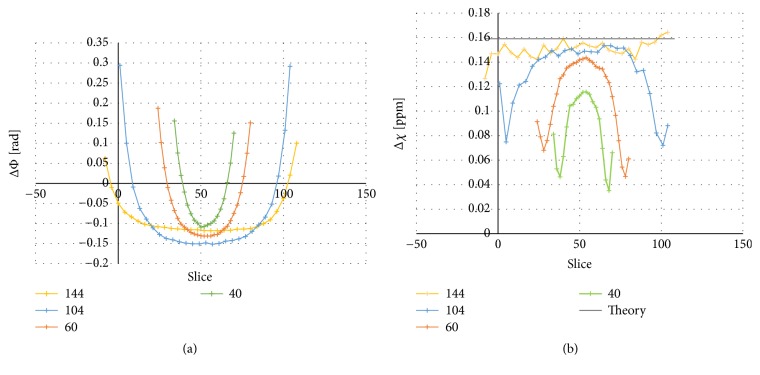
The diagrams show (a) the phase and (b) the QSM-based susceptibility, measured in the largest cylinder of the phantom with different diameters for a varying number of slices: 40 slices correspond to an object coverage of 32 mm, 60 slices correspond to 48 mm, 104 slices correspond to 83.2 mm, and 144 slices correspond to 115.2 mm.

*Volume Coverage*.  Figure 6 shows the result of measuring with different volume coverage. The same slice thickness (0.8 mm) was used for each acquisition.

### 4.5. Variations in Postprocessing and QSM Calculation Procedures

**Figure 7:**
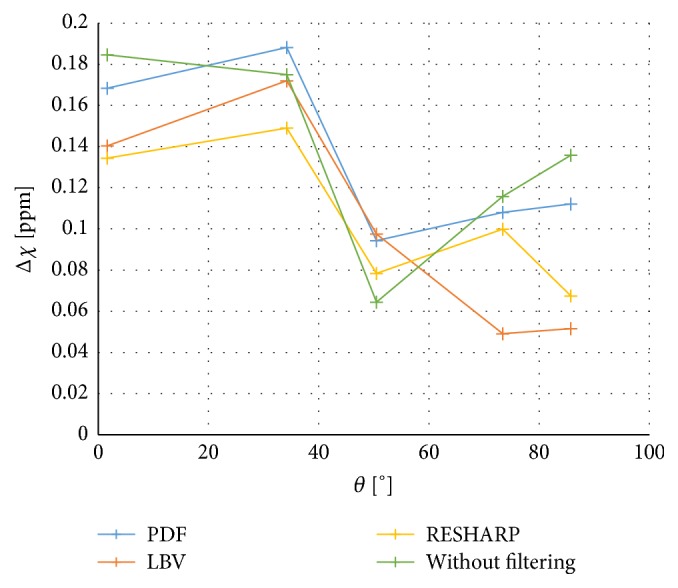
QSM-based magnetic susceptibility in cylinders oriented at different angles relative to the main magnetic field, calculated using three different filtering methods (PDF, LBV, and RESHARP) as well as without filtering.

*Filtering Methods*. Susceptibility estimates obtained using phase data filtered with three different methods for background field removal (PDF, LBV, and RESHARP) as well as without any filtering are presented in Figure 7. 

*Variation of λ*. Although clear differences in QSM image quality were observed for different *λ* settings, the numerical susceptibility values inside cylinders did not vary substantially (approximately ±0.01 ppm from the measured mean value) when *λ* varied between 1 and 50000.

*Zero Padding*. The zero padding did not have any observable effect on the estimated absolute susceptibility values for the phantom with cylinders in various angles relative to the main magnetic field.

### 4.6. Simulations

**Figure 8:**
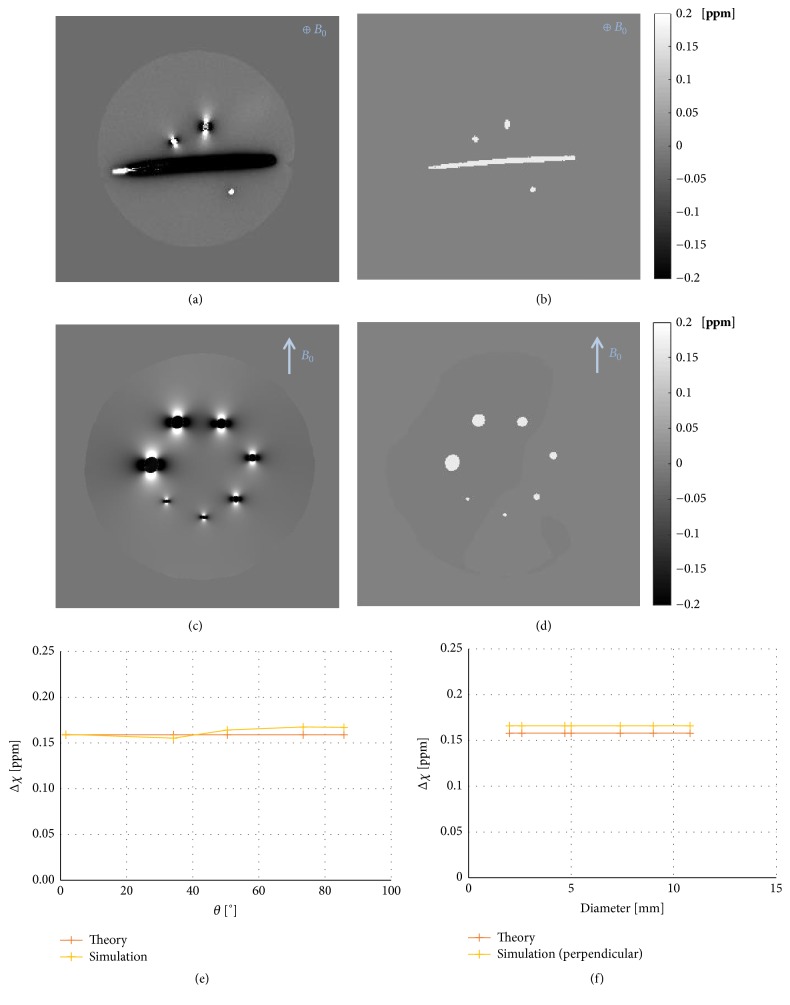
(a) Simulated phase image and (b) the corresponding susceptibility image calculated with MEDI for the phantom with cylinders at different angles (slice 17 of 48). (c) Simulated phase image and (d) the corresponding susceptibility image for the phantom with varying diameters (slice 40 of 80). Images were simulated without added noise for a matrix size of 512×512. (d) Susceptibility registered in cylinders with different angles in simulated images. (e) Calculated susceptibility using simulated phase data, for varying cylinder diameter, compared with the theoretical susceptibility. Simulated cylinders corresponded to 0.5 mM Gd, and the assigned values of magnetic susceptibility in the simulated phantom are given in Table 1.

Simulated phase images and the corresponding artificial susceptibility maps, calculated from simulated phase data using the MEDI algorithm, are shown in Figures 8(a)–8(d). Simulated phase images appeared visually similar to corresponding measured phase images, but, in the simulated QSM images (i.e., calculated from simulated phase maps), no susceptibility dependence on the angle of the cylinder axis relative to the B_0_ field was observed (see Figure 8(e)). In the phantom with varying cylinder diameters, the simulated phase images resulted in susceptibility values that were in much better agreement with theory than the susceptibility values based on measured phase (see Figure 8(f)). These findings can be compared to results from measured data in Figures 2(d) and 4.

### 4.7. Phase Profiles

**Figure 9:**
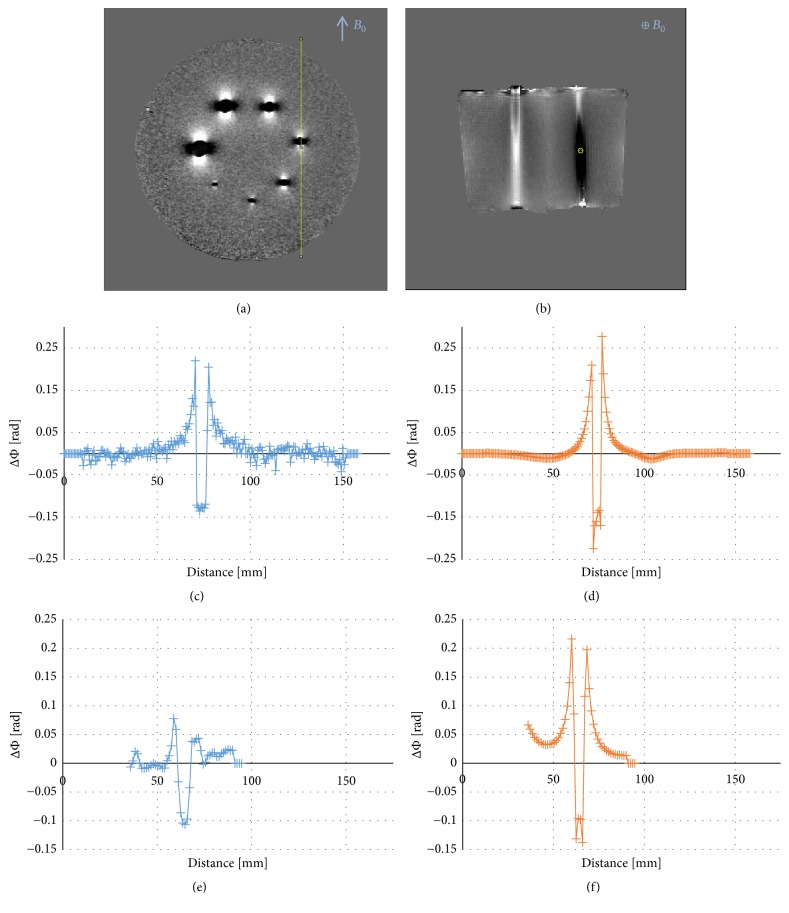
Top row: Positioning of a profile through the 5 mm cylinder perpendicular to the main magnetic field (a) in-plane and (b) along the slice direction. Note: the measurements were identical, except for the use of different slice directions. Middle row: values along the profile (when placed perpendicular to the slice direction) through the 5 mm cylinder in a slice in the middle of the volume showing (c) measured and (d) simulated phase. Bottom row: slice direction phase profile through the 5 mm cylinder; comparison between (e) measured phase and (f) simulated phase.

A profile was positioned through the 5 mm cylinder in measured and simulated phase images of the phantom with cylinders of varying diameters. Profiles were plotted both in-plane and along the slice direction as illustrated in Figures 9(a) and 9(b); i.e., the measurements were identical, except for the use of different slice directions. The results are presented in Figures 9(c)–9(f). Special attention was paid to the amplitude of the peaks at the cylinder edges; no marked difference in peak amplitude was seen between measured and simulated phase for the in-plane profile. However, with the phase profile in the slice direction, the simulated peak phase value was considerably higher than the measured phase.

## 5. Discussion

Cylindrical objects were used in this investigation due to their geometrical resemblance with blood vessels. Also, the distinct angular dependence of the phase in a cylinder (*cf.*Figure 2(a)) made it reasonable to assume that cylinders could be a challenge for the QSM algorithm. Another advantage with the use of cylinders is the availability of theoretical relationships between the local magnetic field change and the magnetic susceptibility, as seen in (3) and (4). In the interpretation of the current results, it should, however, be remembered that cylinders show rather limited resemblance with most* in vivo* structures of relevance to clinical investigations. The initial presumption that cylindrical objects could be problematic for the QSM algorithm seemed, at first, to be valid based on the observation of the measured and theoretical data of the phase and susceptibility inside the cylinders at different angles relative to the main magnetic field (Figure 2(d)). The simulated data, however, indicated that the QSM algorithm did, in fact, generate magnetic susceptibility values that were very close to theory, even for angles larger than the magic angle (*cf.*Figure 8(e)). Furthermore, the simulated data did not show any dependence of estimated susceptibility on the diameter of the phantom (*cf.*Figure 8(f)).

From the measurements on cylinders filled with solutions of varying gadolinium concentrations, it was concluded that the estimated susceptibility varied linearly with the concentration of contrast agent, with an estimated slope of approximately 390 ppm/M using the QSM algorithm and 382 ppm/M using the infinite-cylinder approximation; i.e., the QSM algorithm generated a slightly higher slope than the infinite-cylinder approximation, based on the same experimental phase data. Our current estimates are in good agreement with previous findings for gadoterate meglumine (Dotarem), by Fruytier et al. [42], but higher than the reference (Magnevist) Gd molar susceptibility value of 326 ppm/M [39].

For the 0.5 mM gadolinium solution, also used in the other phantom designs, the susceptibility value was slightly higher than the reference value, in accordance with the results shown in Figure 3(b), as discussed above. Comparing the results of Figures 2(d) and 4 with Figure 3(b) indicates that the deviation of the slope from the reference value (in Figure 3(b)) corresponds well to the slight apparent overestimation of the susceptibility for cylinders approximately parallel to the main magnetic field. Since the phase shift (in Figure 3(a)) and the susceptibility estimates based on the infinite-cylinder approximation (in Figure 3(b)) were also higher than expected from the molar susceptibility reference value, it seems reasonable to conclude that the overestimation was not, at least not entirely, related to the QSM-based susceptibility calculation. The result from the use of simulated phase data confirmed that the problem of estimating the true susceptibility values for different angulations relative to the main magnetic field was not inherent to the MEDI algorithm.

The choice of the regularization parameter *λ* has previously been shown to influence the quantitative accuracy of the QSM method [35]. However, in the present study, the value of *λ* seemed to influence primarily the image quality, but the absolute susceptibility estimates did not vary substantially. The fact that the results were not much affected by the choice of *λ* also suggests that the observed error arises from some other step in the procedure. Hence, three different filtering methods were applied to see if the problems were caused in the background phase removal procedure. Although the results for different filtering methods were not identical, no filtering method returned a systematically more accurate result than the others. An attempt was also made to calculate the susceptibility without filtering. This resulted in images of poor quality, but the angular dependence of the susceptibility estimates remained.

As shown, the phase was measured correctly inside the cylinders but measured and simulated phase data did not generate the same output from the QSM algorithm. Hence, a reasonable conclusion was that measured phase must differ from simulated phase* outside* the cylinder. The comparison of profiles originating from measured and simulated phase showed that the difference in phase between measurement and simulation occurred just outside the cylinder, and the underestimation was more pronounced when phase was recorded along the slice direction. Partial volume effects may have been of importance in this context [43], considering the high spatial phase gradient in this region, and it is not entirely straightforward to predict the exact manifestation of the loss of phase information, occurring in the complex sum of voxel components, in a given imaging situation. Depending on the particular MRI unit and imaging protocol, potential effects of applied deapodization filters might also be relevant to consider in this context. Since the magnetization of the cylinder affects the phase not only inside the cylinder but also in the area surrounding the cylinder, the background phase in the vicinity of the cylinder will influence the result of the QSM susceptibility calculation. Hence, if the phase was not correctly measured in the background region, close to the cylinder, this constitutes a plausible explanation to why QSM calculations failed to return accurate values for the magnetic susceptibility. This explanation is also in accordance with the fact that the infinite-cylinder approximation (see (3)) yielded more reasonable magnetic susceptibility estimates than the QSM algorithm.

In this context, it is also relevant to note that the phantom with parallel cylinders of varying diameters was scanned at two different orientations relative to the main magnetic field (0° and 90°), while keeping the slice orientation orthogonal to the cylinders in each acquisition, i.e., with the measurement of the phase being consistent in terms of slice orientation relative cylinder axis. Comparing the QSM susceptibility results in Figure 4 (for 5 mm diameter) with those in Figure 2(d) from the phantom with cylinders of varying angles (for 0° and 90°) shows that the parallel as well as the perpendicular cylinder orientation yielded very similar QSM susceptibility values between the two separate phantoms (i.e., 0.16-0.17 ppm in the parallel case and 0.10 ppm in the perpendicular case).

Slice spacing, slice thickness, and volume coverage are also important issues in QSM [44]. In our study, a larger volume coverage of the object resulted in susceptibility values closer to the theoretical value and in susceptibility images with a more uniform slice direction profile, in accordance with previous recommendations for an extended spatial coverage in QSM of deep grey matter [45]. Preliminary attempts to remove slices at the edges of the phase image stack, after the PDF but before the MEDI QSM reconstruction, also resulted in images with less artefacts and a more uniform slice direction profile (data not shown), and this suggests that phase values along the slice direction or at the edges of the imaged volume are influenced by factors which are still unknown (e.g., residual slice aliasing effects, etc.).

Furthermore, the spatial resolution seemed to be of some importance for the accuracy of the estimated susceptibility since the estimated susceptibility values appeared to vary between different voxel sizes. For the smallest cylinders (diameters of 2 mm and 2.6 mm) some degree of partial volume effects can be expected, since the voxel size used was 1×1×2 mm^3^, but even for the larger cylinders the estimates differed from the expected values. Figure 5 indicates that there is no dependence on the voxel size for the largest cylinder (10.8 mm in diameter), but for the 5 mm cylinder a higher resolution resulted in a value closer to theory. This implies, not surprisingly, that sufficient spatial resolution is needed to obtain optimal results. At a certain point, the combination of object size and spatial resolution gives sufficiently good measurement conditions, and the systematic error is minimal. For a smaller object, a higher resolution is obviously required to reach that point. However, to establish a clear relationship between image resolution and estimated susceptibility values, more data points would be needed.

Finally, it should be noted that even if experimental phase data were to be accurate, the QSM results would still, in practice, be relative. The assignment of zero phase in the phase maps would imply zero susceptibility, which is normally not the true value for the compartment in question, and, furthermore, the QSM algorithm will typically shift the values of the output data so that the mean value of the volume is close to zero. In the phantoms, the true background susceptibility value is known and thus the expected phase within the cylindrical object can be calculated. In the human body, we do not normally have any such information. Hence, some reference region with known susceptibility is required in order to obtain the correct absolute level of susceptibility within the dataset. CSF has been proposed for such reference purposes, but such an approach is far from straightforward [46].

## 6. Conclusions

The MEDI algorithm was demonstrated to be quite stable for QSM calculations. The choice of parameters and settings, for example, the regularization parameter* λ*, the zero padding, and the choice of filtering method seemed not to have a large impact on the quantitative results. Most importantly, when applied to simulated phase maps, MEDI returned accurate susceptibility quantification also for challenging geometries. For experimental phase data, the QSM algorithm did result in a linear relationship between susceptibility and concentration of contrast agent, but correct susceptibility was not obtained for cylindrical objects at an angle close to or larger than the magic angle. The error seemed to originate from the phase measurement rather than from imperfections in the QSM susceptibility calculation. In our MRI installation, deviation from theory was observed primarily along the slice direction, in the phantom background (i.e., gel) region of the measured phase images. The QSM-based susceptibility results seemed to be somewhat more accurate for large objects and/or good spatial resolution, large volume coverage of the object, and with the slice direction applied along the long axis of the object of interest.

## Figures and Tables

**Table 1:** Reference magnetic susceptibility values of the different components of the phantoms.

**χ**(**water**)	-9.022 ppm
**χ**_**mol**_(**Ni**(**NO**_3_)_2_ · 6**H**_2_**O**)	54 ppm/M
**χ**_**mol**_(**Gd**)	326 ppm/M in reference to water
**χ**(**gel**)	-9.017 ppm
**χ**(0.5 **mM** **Gd**)	-8.859 ppm

**Table 2:** Imaging parameters in the standard protocol used for QSM phase measurements.

**Sequence**	3D Multi-TE Gradient Echo
**Number of echoes**	11
**Echo spacing, **Δ**TE**	6.78 ms
**Flip angle**	20°
**Band width**	150 Hz/pixel
**Field of view, FOV**	24 cm
**Slice thickness **	2 mm
**Number of averages**	1
**Matrix size**	256 x 240

## Data Availability

Requests for data will be considered by the corresponding author.

## References

[1] Liu S., Buch S., Chen Y. (2017). Susceptibility-weighted imaging: current status and future directions. *NMR in Biomedicine*.

[2] Li L., Leigh J. S. (2004). Quantifying arbitrary magnetic susceptibility distributions with MR. *Magnetic Resonance in Medicine*.

[3] Shmueli K., de Zwart J. A., van Gelderen P., Li T.-Q., Dodd S. J., Duyn J. H. (2009). Magnetic susceptibility mapping of brain tissue in vivo using MRI phase data. *Magnetic Resonance in Medicine*.

[4] Wharton S., Schäfer A., Bowtell R. (2010). Susceptibility mapping in the human brain using threshold-based k-space division. *Magnetic Resonance in Medicine*.

[5] Yablonskiy D. A., Sukstanskii A. L. (2017). Effects of biological tissue structural anisotropy and anisotropy of magnetic susceptibility on the gradient echo MRI signal phase: theoretical background. *NMR in Biomedicine*.

[6] Lim I. A. L., Faria A. V., Li X. (2013). Human brain atlas for automated region of interest selection in quantitative susceptibility mapping: Application to determine iron content in deep gray matter structures. *NeuroImage*.

[7] Zhang Y., Wei H., Cronin M. J., He N., Yan F., Liu C. (2018). Longitudinal atlas for normative human brain development and aging over the lifespan using quantitative susceptibility mapping. *NeuroImage*.

[8] Stüber C., Pitt D., Wang Y. (2016). Iron in multiple sclerosis and its noninvasive imaging with quantitative susceptibility mapping. *International Journal of Molecular Sciences*.

[9] Stankiewicz J., Panter S. S., Neema M., Arora A., Batt C. E., Bakshi R. (2007). Iron in chronic brain disorders: imaging and neurotherapeutic implications. *Neurotherapeutics*.

[10] Kudo K., Liu T., Murakami T. (2016). Oxygen extraction fraction measurement using quantitative susceptibility mapping: Comparison with positron emission tomography. *Journal of Cerebral Blood Flow & Metabolism*.

[11] Zhang J., Liu T., Gupta A., Spincemaille P., Nguyen T. D., Wang Y. (2015). Quantitative mapping of cerebral metabolic rate of oxygen (CMRO_2_) using quantitative susceptibility mapping (QSM). *Magnetic Resonance in Medicine*.

[12] Duyn J. H., Schenck J. (2017). Contributions to magnetic susceptibility of brain tissue. *NMR in Biomedicine*.

[13] Wirestam R. (2012). Using contrast agents to obtain maps of regional perfusion and capillary wall permeability. *Imaging in Medicine*.

[14] Bonekamp D., Barker P. B., Leigh R., Van Zijl P. C. M., Li X. (2015). Susceptibility-based analysis of dynamic gadolinium bolus perfusion MRI. *Magnetic Resonance in Medicine*.

[15] Xu B., Spincemaille P., Liu T. (2015). Quantification of cerebral perfusion using dynamic quantitative susceptibility mapping. *Magnetic Resonance in Medicine*.

[16] Xie X. L., Layton A. T., Wang N. (2016). Dynamic contrast-enhanced quantitative susceptibility mapping with ultrashort echo time MRI for evaluating renal function. *American Journal of Physiology-Renal Physiology*.

[17] Langkammer C., Schweser F., Shmueli K. (2018). Quantitative susceptibility mapping: Report from the 2016 reconstruction challenge. *Magnetic Resonance in Medicine*.

[18] Bao L., Li X., Cai C., Chen Z., Van Zijl P. C. M. (2016). Quantitative susceptibility mapping using structural feature based collaborative reconstruction (SFCR) in the human brain. *IEEE Transactions on Medical Imaging*.

[19] Wang Y., Liu T. (2015). Quantitative susceptibility mapping (QSM): decoding MRI data for a tissue magnetic biomarker. *Magnetic Resonance in Medicine*.

[20] Zhou D., Cho J., Zhang J., Spincemaille P., Wang Y. (2017). Susceptibility underestimation in a high-susceptibility phantom: Dependence on imaging resolution, magnitude contrast, and other parameters. *Magnetic Resonance in Medicine*.

[21] Langham M. C., Magland J. F., Epstein C. L., Floyd T. F., Wehrli F. W. (2009). Accuracy and precision of MR blood oximetry based on the long paramagnetic cylinder approximation of large vessels. *Magnetic Resonance in Medicine*.

[22] Cronin M. J., Wang N., Decker K. S., Wei H., Zhu W.-Z., Liu C. (2017). Exploring the origins of echo-time-dependent quantitative susceptibility mapping (QSM) measurements in healthy tissue and cerebral microbleeds. *NeuroImage*.

[23] Hsieh C.-Y., Cheng Y.-C. N., Neelavalli J., Haacke E. M., Stafford R. J. (2015). An improved method for susceptibility and radius quantification of cylindrical objects from MRI. *Magnetic Resonance Imaging*.

[24] Xie H., Cheng Y.-C. N., Kokeny P. (2016). A quantitative study of susceptibility and additional frequency shift of three common materials in MRI. *Magnetic Resonance in Medicine*.

[25] Li J., Chang S., Liu T. (2012). Reducing the object orientation dependence of susceptibility effects in gradient echo MRI through quantitative susceptibility mapping. *Magnetic Resonance in Medicine*.

[26] Olsson E. (2016). *MRI-Based Quantification of Magnetic Susceptibility: Assessment of Measurement And Calculation Accuracy (Unpublished MSc Dissertation)*.

[27] Cusack R., Papadakis N. (2002). New robust 3-D phase unwrapping algorithms: application to magnetic field mapping and undistorting echoplanar images. *NeuroImage*.

[28] Liu T., Khalidov I., de Rochefort L. (2011). A novel background field removal method for MRI using projection onto dipole fields (PDF). *NMR in Biomedicine*.

[29] de Rochefort L., Liu T., Kressler B. (2010). Quantitative susceptibility map reconstruction from MR phase data using bayesian regularization: validation and application to brain imaging. *Magnetic Resonance in Medicine*.

[30] Zhou D., Liu T., Spincemaille P., Wang Y. (2014). Background field removal by solving the Laplacian boundary value problem. *NMR in Biomedicine*.

[31] Sun H., Wilman A. H. (2014). Background field removal using spherical mean value filtering and Tikhonov regularization. *Magnetic Resonance in Medicine*.

[32] Salomir R., De Senneville B. D., Moonen C. T. W. (2003). A fast calculation method for magnetic field inhomogeneity due to an arbitrary distribution of bulk susceptibility. *Concepts in Magnetic Resonance B*.

[33] Marques J. P., Bowtell R. (2005). Application of a Fourier-based method for rapid calculation of field inhomogeneity due to spatial variation of magnetic susceptibility. *Concepts in Magnetic Resonance*.

[34] Liu T., Wisnieff C., Lou M., Chen W., Spincemaille P., Wang Y. (2013). Nonlinear formulation of the magnetic field to source relationship for robust quantitative susceptibility mapping. *Magnetic Resonance in Medicine*.

[35] Liu J., Liu T., de Rochefort L. (2012). Morphology enabled dipole inversion for quantitative susceptibility mapping using structural consistency between the magnitude image and the susceptibility map. *NeuroImage*.

[36] Liu T., Liu J., De Rochefort L. (2011). Morphology enabled dipole inversion (MEDI) from a single-angle acquisition: Comparison with COSMOS in human brain imaging. *Magnetic Resonance in Medicine*.

[37] Christoffersson J. O., Olsson L. E., Sjöberg S. (1991). Nickel-doped agarose gel phantoms in MR imaging. *Acta Radiologica*.

[38] Pople J. A., Schneider W. G., Bernstein H. J. (1959). *High-Resolution Nuclear Magnetic Resonance*.

[39] De Rochefort L., Brown R., Prince M. R., Wang Y. (2008). Quantitative MR susceptibility mapping using piece-wise constant regularized inversion of the magnetic field. *Magnetic Resonance in Medicine*.

[40] Cornell MRI Research Lab http://weill.cornell.edu/mri/pages/qsm.html.

[41] Kressler B., de Rochefort L., Liu T., Spincemaille P., Jiang Q., Wang Y. (2010). Nonlinear regularization for per voxel estimation of magnetic susceptibility distributions from MRI field maps. *IEEE Transactions on Medical Imaging*.

[42] Fruytier A.-C., Magat J., Colliez F., Jordan B., Cron G., Gallez B. (2014). Dynamic contrast-enhanced MRI in mice at high field: Estimation of the arterial input function can be achieved by phase imaging. *Magnetic Resonance in Medicine*.

[43] Ward P. G., Fan A. P., Raniga P. (2017). Improved quantification of cerebral vein oxygenation using partial volume correction. *Frontiers in Neuroscience*.

[44] Karsa A., Biondetti E., Punwani S., Shmueli K. The effect of large slice thickness and spacing and low coverage on the accuracy of susceptibility mapping.

[45] Elkady A. M., Sun H., Wilman A. H. (2016). Importance of extended spatial coverage for quantitative susceptibility mapping of iron-rich deep gray matter. *Magnetic Resonance Imaging*.

[46] Lind E., Knutsson L., Kämpe R., Ståhlberg F., Wirestam R. (2017). Assessment of MRI contrast agent concentration by quantitative susceptibility mapping (QSM): application to estimation of cerebral blood volume during steady state. *Magnetic Resonance Materials in Physics, Biology and Medicine*.

